# The Correlation Between Hearing Loss, Especially High-Frequency Hearing Loss and Cognitive Decline Among the Elderly

**DOI:** 10.3389/fnins.2021.750874

**Published:** 2021-11-15

**Authors:** Tongxiang Diao, Xin Ma, Junbo Zhang, Maoli Duan, Lisheng Yu

**Affiliations:** ^1^Department of Otolaryngology, Head and Neck Surgery, People’s Hospital, Peking University, Beijing, China; ^2^Department of Otolaryngology, Head and Neck Surgery, Peking University First Hospital, Beijing, China; ^3^Department of Clinical Science, Intervention and Technology, Karolinska Institute, Stockholm, Sweden; ^4^Department of Otolaryngology, Head and Neck Surgery & Audiology and Neurotology, Karolinska University Hospital, Karolinska Institute, Stockholm, Sweden

**Keywords:** hearing loss, high-frequency hearing loss, cognition, language ability, abstract ability

## Abstract

**Objective:** The relation between cognition and hearing loss has been increasingly paid high attention, however, few studies have focused on the role of high-frequency hearing loss in cognitive decline. This study is oriented to role of hearing loss especially high-frequency hearing loss in cognitive impairment among elderly people (age ≥ 60 years).

**Methods:** The Montreal Cognitive Assessment Scale (MoCA) and pure tone audiometry were used to investigate the hearing loss and cognitive function of 201 elderly people older than 60 years. Factors possibly related to cognitive impairment including age, years of education, occupation, living conditions, history of otologic diseases, and high blood pressure were registered. This study consisted of two parts. First, univariate analysis and multiple linear regressions were performed to analyze the possible influencing factors of cognitive function among the 201 elderly people. Second, average hearing thresholds of low frequencies (250, 500 Hz), intermediate frequencies (1 k, 2 kHz), and high frequencies (4 k, 8 kHz) were calculated to screen out 40 cases with high-frequency hearing loss alone and 18 cases with normal hearing. Univariate analysis was used to compare the general condition, cognitive function, and each cognitive domain between the two groups, analyzing the relation between high-frequency hearing loss and cognitive function.

**Result:** We found that age, years of education, pure tone average (PTA), occupation, living condition, history of otologic diseases, years of self-reported hearing loss, and hypertension history were related to cognitive function. Furthermore, age, education experience, duration of self-reported hearing loss, and hypertension were independent factors (*p* < 0.05). PTA was negatively related with attention, orientation, and general cognition (*p* < 0.05). There were only 18 cases (9.0%) with normal hearing, and 40 cases (19.9%) with abnormal high-frequency hearing alone. The overall cognitive function showed no significant difference between them (*p* > 0.05); in contrast, the speech and abstract ability were significantly decreased in cases with high-frequency hearing loss (*p* < 0.05).

**Conclusion:** The increase of PTA among the elderly may affect the overall cognition by reducing attention and orientation. High-frequency hearing loss alone can affect the language and abstract ability to a certain extent, which is worthy of more attention.

## Introduction

Hearing loss has become the third leading disability factor in the worldwide according to the latest researches, which is an important factor affecting human health, especially the health of the elderly (GBD 2016 Disease and Injury Incidence and Prevalence Collaborators, 2017). In recent years, the correlation between hearing loss and cognitive decline has attracted more and more attention. Numerous studies, including several systematic review articles and meta-analyses, have shown that hearing loss is remarkably related with cognitive dysfunction, impaired performance of various cognitive domains, and the occurrence of dementia (Thomson et al., 2017; Zheng et al., 2017; Ford et al., 2018; Loughrey et al., 2018). Cognitive impairment includes memory, learning, orientation, comprehension, judgment, calculation, language, visuospatial, analysis and problem-solving ability and is often accompanied by mental, behavioral, and personality abnormalities at a certain stage of the disease process (Arlington, 2013). A meta-analysis conducted by Loughrey et al. (2018) found that there was a significant association between age-related hearing loss (ARHL) and cognitive impairment [odds ratio, 1.22; 95% confidence interval (CI), 1.09–1.36] among the prospective cohort studies and a small but statistically significant association between ARHL and seven cognitive domains, such as episodic memory and processing speed, among the cohort studies (Loughrey et al., 2018). Meanwhile, a novel lifespan-based model of dementia risk was reported by the Lancet Commission on Dementia Prevention, Intervention, and Care and simultaneously published in *Lancet* at the 2017 Alzheimer’s Association International Conference in London (Livingston et al., 2017). Hearing loss was positioned as the largest potentially modifiable risk factor for dementia among nine health and lifestyle factors. The Lancet Commission found that midlife hearing loss, if eliminated, might reduce the risk of dementia by 9%. The underlying causal mechanisms leading to the connection between the two are not well understood. Several possible relationships have been postulated. (1) cognitive load on perception hypothesis (cognitive decline may reduce the cognitive resources that are available for auditory perception, manifesting as hearing loss) (Lin et al., 2013); (2) sensory-deprivation hypothesis (hearing loss causes cognitive decline that is permanent) (Lindenberger and Baltes, 1994); (3) information-degradation hypothesis (hearing loss causes cognitive decline which is potentially remediable) (Pichora-Fuller, 2003); and (4) common cause hypothesis (a third factor causes both declines) (Baltes and Lindenberger, 1997).

However, most previous studies have focused on the correlation between speech-frequency hearing loss and cognitive function, while ignoring the importance of high-frequency hearing loss. Studies have shown that the incidence of high-frequency hearing loss is significantly higher than that of speech frequency (REF). Among people aged 20 to 29 years, the proportion of combined speech-frequency hearing loss is only 2.2%, whereas the proportion of high-frequency hearing loss can be as high as 7% (Hoffman et al., 2017). And this difference is more obvious in the elderly group. Among people aged 60 to 69 years, the incidence of speech-frequency hearing loss is 39.3%, and the proportion of high-frequency hearing loss can be as high as 68% (Hoffman et al., 2017). These data indicate that high-frequency hearing tends to decline earlier than speech-frequency hearing. Previously, it was believed that high-frequency hearing loss would not affect the daily life of the patients significantly. However, with the introduction of hidden hearing loss, the important role of high-frequency hearing in the speech recognition has attracted more and more attention (Monson et al., 2019). Some studies have already shown that high-frequency hearing is key for speech recognition, especially speech perception in noise (George et al., 2007; Motlagh Zadeh et al., 2019; Varnet et al., 2019). However, there is no study that has focused on the correlation between pure high-frequency hearing loss and cognitive function except for speech perception. This study chose the elderly as the subjects and studied the correlation between average of hearing loss, especially the degree of pure high-frequency hearing loss, and cognitive function to explore the relationship between hearing loss and cognitive decline and the underline mechanism, providing a theoretical basis for early detection and prevention of senile cognitive decline in clinical practice.

## Materials and Methods

### Participants

A total of 201 elderly volunteers were enrolled in this study from the free clinic of 2019 Ear Day at Peking University People’s Hospital. The detailed inclusion criteria were as follows: (1) age ≥ 60 years, (2) sensorineural hearing loss, and no significant difference in hearing threshold between two ears, (3) no history of otitis media, sudden deafness history, and acoustic neuroma, and (4) no mental illness history and could cooperate with the whole study process. All study subjects underwent pure tone audiometry test, otoscope examination, and cognitive function assessment. The basic information including sex, age, living condition, years of education, occupation, self-reported hearing loss, tinnitus, ear diseases history, and some chronic disease history (hypertension, diabetes, and hyperlipidemia) was collected by face-to-face survey with questionnaires. All included subjects and (or) their family members were asked to fill the questionnaire under the supervision of the same experienced physician.

### Study Design

A questionnaire on cognitive function and influencing factors was designed according to previous study (Hugo and Ganguli, 2014). All the elderly participants were examined during the Ear Day free clinic. When the survey was completed, the questionnaires and related examination data were returned.

### Cognitive Function Assessment

The cognitive function assessments of all participants were completed with a unified Montreal Cognitive Assessment Scale (MoCA) questionnaire survey, which was administered to assess the global cognitive level by the same neurologist. The MoCA scale consisted of eight several subtasks to assess different cognitive domains, of which the immediate memory subdomain is not scored. The other seven cognitive domains and corresponding scores were visuospatial and executive function for 5 points, language ability for 3 points, attention for 6 points, orientation for 6 points, delayed memory for 5 points, naming for 3 points, and abstract ability for 2 points (Nasreddine et al., 2005). Moreover, existing studies have shown that the scores of different cognitive domains of MoCA assessment result may represent the different cognitive function (Faria et al., 2020; Rossetto et al., 2020).

### Auditory and Otoscope Tests

The pure tone audiometry and the otoscope examination were all completed in the ENT departments of Peking University People’s Hospital. According to the World Health Organization criteria (1997), the average value of 500−, 1, 000−, 2, 000−, and 4,000-Hz air conduction pure tone average (PTA) hearing thresholds was used for defining the hearing loss degree, including normal hearing (≤25 dB), mild (26–40 dB), moderate (41–60 dB), severe (61–80 dB), and profound hearing loss (>80 dB). On the other hand, the average hearing thresholds of low frequencies (250, 500 Hz), intermediate frequencies (1 k, 2 kHz), and high frequencies (4 k, 8 kHz) were all calculated separately to select a group of subjects with hearing loss at only high frequencies (4 k, 8 kHz) (HHL group). Except for average hearing threshold of 4 and 8 k is greater than 25 dB, the hearing thresholds of other frequencies (250 Hz, 500 Hz, 1 k, 2 k) of patients in the HHL group are all not higher than 25 dB (HHL group).

### Ethics Statement

The Peking University People’s Hospital Ethical permission committee approved study (2019PHB084-01), and all subjects provided their informed consents.

### Statistical Analysis

Statistical analyses in this study were performed with SPSS 24.0 software package (IBM, Armonk, NY, United States). This study consisted of two parts. First, univariate analysis and multiple linear regressions were performed to analyze the possible influencing factors of cognitive function among the 201 elderly people. Second, average hearing thresholds of low frequencies (250, 500 Hz), intermediate frequencies (1 k, 2 kHz), and high frequencies (4 k, 8 kHz) were calculated to screen out 40 cases with high-frequency hearing loss alone (HHL) and 18 cases with absolutely normal hearing (NH). Univariate analysis was used to compare the general condition, cognitive function, and each cognitive domain between the two groups, analyzing the relation between high-frequency hearing loss and cognitive function. All the statistical significances were at the level *p* < 0.05.

## Results

### Epidemiology and Clinical Characteristics of All the Participants

There were 201 participants (101 females, 50.2%; 100 males, 49.8%). The overall average age was 72.06 ± 5.90 years, with a range of 60 to 90 years. According to PTA values, the number of participants with normal hearing, mild, moderate, severe, and profound hearing loss were 39 (19.4%), 65 (32.3%), 80 (39.8%), 17 (8.5%), and 0 (0%), respectively. The overall average score of MoCA scale assessment was 24.64 ± 3.68, with a range of 11 to 30.

### Factors Related to Cognitive Function in the Elderly

As shown in Table 1, the univariate analysis showed that age, education experience, occupation, living conditions, ear diseases history, hypertension, PTA, and years of self-reported hearing loss remarkably correlated with the results of cognitive assessment (*p* < 0.05). The above factors were all included in a multivariate analysis, which indicated that age, years of education, years of self-reported hearing loss, and hypertension were independent factors related to cognitive function (*p* < 0.05). The elderly with older age, shorter years of education, longer time of self-conscious hearing loss, and high blood pressure were more likely to be associated with cognitive dysfunction.

**TABLE 1:** Factors related to cognitive function among the elderly.

Univariable analysis			

Variable	Number (percentage)	Cognitive function ($\bar{\text{x}}$ ± μ)	*p*
Sex (female)	101/201 (50.2%)	24.30 ± 3.80	0.139
Age (years)	201	−0.116 (−0.201, −0.030)	0.008*
Occupation			0.027*
Physical	137/201 (68.16%)	23.797 ± 4.0832	
Mental	64/201 (31.84%)	25.029 ± 3.4170	
Education experience (years)	201	0.417 (0.262, 0.572)	0.000*
Living situation			
Living with spouse	158/201 (78.61%)	25.006 ± 3.2644	0.028*
Others	43/201 (21.39%)	23.279 ± 4.7073	
Ear disease history	153/201 (76.1%)	24.3 ± 3.9	
No	37/201 (18.4%)	25.8 ± 2.8	0.044*
Otitis media	11/201 (5.5%)	25.7 ± 2.4	
Sudden deafness			
Hypertension	96/201 (47.8%)	24.000 ± 3.8906	0.022*
Diabetes	64/201 (31.84%)	24.094 ± 4.0147	0.153
Hyperlipidemia	99/201 (49.25%)	24.354 ± 3.5637	0.283
PTA			
≤25 dBHL	39/201 (19.40%)	39 25.051 ± 2.5950	
26–40 dBHL	65/201 (32.34%)	65 25.292 ± 3.7279	
41–60 dBHL	80/201 (39.80%)	80 24.375 ± 3.8397	0.016*
61–80 dBHL	17/201 (8.46%)	17 22.235 ± 4.0083	
Self-reported hearing loss	169/201 (84.08%)	24.497 ± 3.7783	0.270
Duration of self-reported hearing loss (years)			
≤5 y	153/201 (76.12%)	24.908 ± 3.4724	0.048*
>5 y	48/201 (23.88%)	23.708 ± 4.1767	

**Multivariable analysis**			

**Variable**	***B***	**95% CI**	***p***

Age	−0.191	(−0.271, −0.111)	0.000*
Education experience (years)	0.512	(0.361, 0.663)	0.000*
Duration of self-reported hearing loss (years)	−1.164	(−2.218, −0.111)	0.030*
Hypertension	−0.990	(−1.895, −0.086)	0.032*

* Statistical significance.

### Correlation Between Pure Tone Average and Different Cognitive Domains Among the Elderly

Among the elderly people with different hearing levels, the comparisons of cognitive functions suggested that abstract and orientation abilities, and overall cognitive function all deteriorate with the increasing hearing loss degrees (*p* < 0.05). No other cognitive domains showed significant differences between these groups (*p* > 0.05) (Table 2).

**TABLE 2:** Correlation between PTA and different cognitive domains among the elderly.

	PTA				*p*
	≤25 dBHL *n* = 39	26 ∼ 40 dBHL *n* = 65	41 ∼ 60 dBHL *n* = 80	61 ∼ 80 dBHL *n* = 17	
Visual spatial and executive function	4.256 ± 0.8801	4.246 ± 1.1596	4.100 ± 1.0626	3.765 ± 1.0326	0.344
Naming	2.795 ± 0.4091	2.800 ± 0.4402	2.863 ± 0.4132	2.882 ± 0.3321	0.711
Attention	5.436 ± 0.9678	5.477 ± 0.8680	5.375 ± 0.8624	5.235 ± 1.1472	0.771
Language	2.231 ± 0.7420	2.169 ± 0.7618	1.988 ± 0.9345	1.941 ± 1.2976	0.384
Abstract	1.667 ± 0.5298	1.554 ± 0.6381	1.525 ± 0.6931	0.882 ± 0.7812	0.001*
Delayed memory	2.667 ± 1.3443	3.138 ± 1.4018	2.663 ± 1.7059	2.294 ± 1.7594	0.125
Orientation	6.000 ± 0.0000	5.908 ± 0.3411	5.863 ± 0.4132	5.235 ± 1.2005	0.000*
Total	25.051 ± 2.5950	25.29 ± 3.7279	24.375 ± 3.8397	22.235 ± 4.0083	0.016*

* Statistical significance.

### Clinical Characteristics and Cognitive Function of Subjects in HHL Group

**FIGURE 1:**
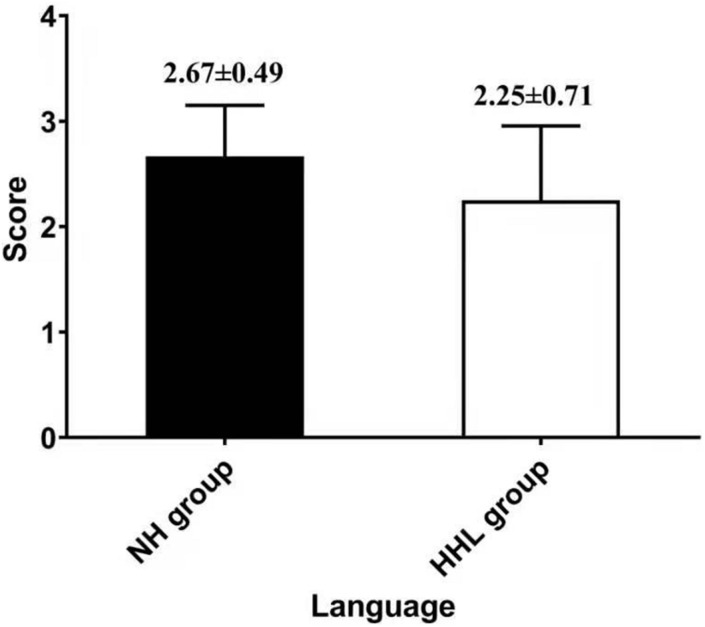
Differences in language ability between the HHL and NH groups.

**FIGURE 2:**
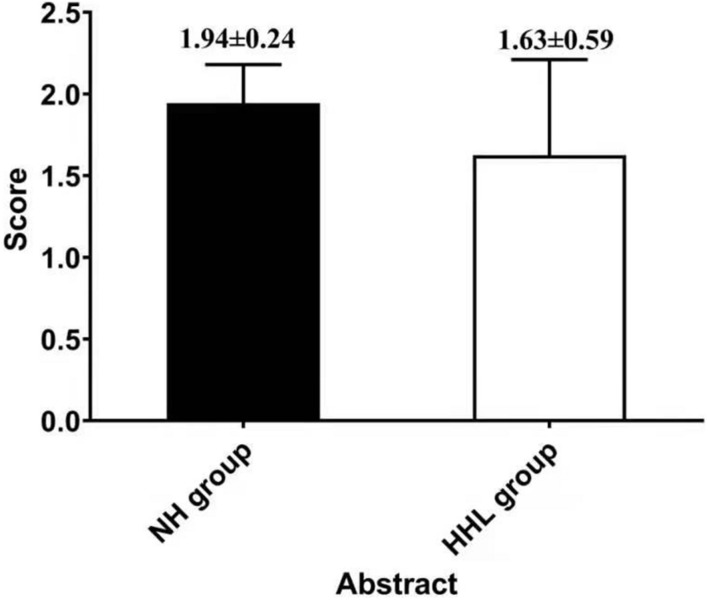
The difference in abstract ability between the HHL and NH groups.

There were only 18 subjects (9.0%) with completely normal hearing levels (<25 dB) at all frequencies tested, including 0.25, 0.5, 1.0, 2.0, 3.0, 4.0, and 8.0 kHz (normal hearing group, NH group). On the other hand, the number of subjects with hearing loss (>25 dB) at only high frequencies (4.0 k and 8.0 kHz) was 40 (19.9%) (high-frequency hearing loss group, HHL group). As shown in Table 3, the overall PTA and the PTA at high frequencies (PTA-HF) were both significantly higher in the HHL group than in the NH group (*p* = 0.013 vs. *p* < 0.001). Meanwhile, no other basic information we studied showed significant differences between these two groups (*p* > 0.05). As shown in Table 4 and Figures 1, 2, the comparisons of cognitive function between these two groups suggested that the language and abstract ability scores were both significantly lower in the HHL group than in the NH group (*p* = 0.027 vs. *p* = 0.005). Meanwhile, no significant differences were found in other cognitive domains and overall cognitive function (*p* > 0.05).

**TABLE 3:** The epidemiology and clinical characteristics of the HHL group and NH groups.

	Total (*n* = 58)	NH (*n* = 18)	HHL (*n* = 40)	*p*
Sex (female)	35	13/35 (37.14%)	22/35 (62.86%)	0.215
Age (years)	69.78 ± 2.96	69.17 ± 3.45	70.05 ± 2.72	0.297
PTA	21.96 ± 6.71	18.75 ± 7.30	23.41 ± 5.96	0.013*
PTA-HF	36.51 ± 13.92	21.81 ± 8.04	43.13 ± 10.50	< 0.001*
Living situation				0.215
Living with spouse	8	1/8 (12.5%)	7/8 (87.5%)	
Others	50	17/50 (34.00%)	33/50 (66.00%)	
Education experience (years)	13.34 ± 2.91	13.06 ± 3.72	13.48 ± 2.52	0.667
Occupation				0.282
Physical	17	7/17 (41.18%)	10/17 (58.82%)	
Mental	40	10/40 (25.00%)	30/40 (75.00%)	
Self-reported hearing loss	22	8/22 (36.36%)	14/22 (63.64%)	0.493
Duration of self-reported hearing loss (years)				1.000
≤5y	52	16/52 (30.77%)	36/52 (69.23%)	
>5y	6	2/6 (33.33%)	4/6 (66.67%)	
Tinnitus	23	8/23 (34.78%)	15/23 (65.22%)	0.617
Hypertension	29	12/29 (41.38%)	17/29 (58.62%)	0.089
Diabetes	19	5/19 (26.32%)	14/19 (73.68%)	0.588
Hyperlipidemia	31	9/31 (29.03%)	22/31 (70.97%)	0.724

* Statistical significance.

**TABLE 4:** Cognitive function and different cognitive domains of the HHL and NH groups.

	Total (*n* = 58)	NH (*n* = 18)	HHL (*n* = 40)	*p*
Visual spatial and executive function	4.40 ± 0.82	4.44 ± 0.62	4.38 ± 0.90	0.767
Naming	2.79 ± 0.45	2.89 ± 0.32	2.75 ± 0.49	0.209
Attention	5.52 ± 0.86	5.56 ± 0.71	5.50 ± 0.93	0.823
Language	2.38 ± 0.67	2.67 ± 0.49	2.25 ± 0.71	0.027*
Abstract	1.72 ± 0.52	1.94 ± 0.24	1.63 ± 0.59	0.005*
Delayed memory	2.95 ± 1.29	2.78 ± 1.31	3.03 ± 1.29	0.504
Orientation	5.97 ± 0.18	6.00 ± 0.00	5.95 ± 0.22	0.160
Total	25.69 ± 2.64	26.17 ± 2.31	25.48 ± 2.78	0.361

* Statistical significance.

## Discussion

As the aging problem increasing worldwide, hearing loss as one of the most common problems among the elderly has drawn more and more attention. Studies have shown that hearing loss is a modifiable age-associated condition linked to dementia (Livingston et al., 2017); however, the underlying mechanism of the association between hearing loss and cognitive decline remains unclear. Of all the 201 elderly participants in this study, only 18 had completely normal hearing. Among the remaining 183 participants, 143 had speech-frequency hearing loss (78.14%), and 40 had only high-frequency hearing loss (21.86%). In this study, we found that the hearing loss is related but not independent influencing factor of cognitive decline, which mainly affects cognitive function by affecting abstract and orientation ability. The age, years of education, years of self-reported hearing loss, and hypertension are independent factors related to the cognitive function of the elderly. In addition, although pure high-frequency hearing loss had no remarkable relevance with general cognitive function, it affected abstract and speech ability.

### Related Factors of Cognitive Function Among the Elderly

The results of this study indicated that among the elderly, occupation, living conditions, ear diseases history, and PTA were the influencing but not independent influencing factors of cognitive function, whereas age, educational experience, years of self-reported hearing loss, and whether accompanied by hypertension were independent related factors of cognitive function. Among them, age is unchangeable, hypertension can be regulated by tertiary prevention, the years of education requires the joint efforts of the whole society, whereas the years of self-reported hearing loss is the only factor that can be changed by early hearing interventions.

As early as 2001, a clinical study conducted by Gomez et al. (2001) proposed that the degree of self-conscious hearing loss could be used as a simple way to self-evaluation of hearing level. Amieva et al. (2015) also proposed that self-reported hearing loss was an independent factor of the cognitive decline among the elderly, and the use of hearing aids can slow down the process of cognitive decline. From the behavior perspective, a number of elderly people with hearing loss often choose to avoid social activities because of the impact of hearing loss on communication, which leads to social isolation and coexistence of the decrease in communication between family and friends eventually leading to depression or cognitive impairment (Brink and Stones, 2007; Mick et al., 2014; Pichora-Fuller et al., 2015). From the neurological perspective, on the one hand, chronic hearing loss will lead to a decrease in the activity of the central auditory system, trigger the compensation mechanism, and ultimately lead to the associated disorder of the auditory center-limbic system and the disuse atrophy of the frontal lobe. On the other hand, hearing loss will cause the reduction of the cognitive capacity, which mainly represents the ability to minimize pathological damage by cognitive pregeneration processes or activation of compensatory mechanisms (Stern, 2002; Tun et al., 2009) in the central system. As theorized by the cognitive load hypothesis, that hearing loss leads to degraded auditory signals, greater cognitive resources being required for auditory perceptual processing, and diversion from other cognitive tasks to effortful listening, eventually resulting in cognitive reserve depletion (Tun et al., 2009).

However, there are several researchers who believe that the association between hearing loss and cognitive impairment is caused by overestimation, namely, overdiagnosis hypothesis, in which degraded hearing, rather than cognitive function, impacts performance on certain neuropsychological tests. First, despite that hearing loss usually occurs earlier than dementia or cognitive impairment, suggesting hearing loss may lead to dementia or cognitive impairment, it may also be due to the fact that patients with hearing loss usually receive more neuropsychological tests. Therefore, it is easier to diagnose the accompanying cognitive impairment (Fulton et al., 2015). Or more broadly speaking, hearing loss itself can cause bias in neuropsychological evaluations, because most neuropsychological evaluations need to be achieved through speech. Furthermore, as verbal instructions or tasks that rely considerably on hearing are used during cognitive assessments, individuals with hearing difficulty are sometimes at a disadvantage. The selection of tests for cognitive measures that are heavily loaded for verbal skills is not appropriate for individuals with hearing difficulty; however, even when the response mode of a measure is non-verbal, instructions for tasks can be complex or difficult to perceive for the hearing-impaired. Any degree of hearing loss can affect functioning and test performance for neuropsychological assessments (Hill-Briggs et al., 2007; Dupuis et al., 2015; Jorgensen et al., 2016).

### Correlation Between Hearing Loss and Cognitive Function Among the Elderly

In this study, we found that hearing loss has significantly related to the cognitive decline, which is consistent with the previous study conducted by Lin et al. (2011), They found the annual hearing threshold of participants with dementia was higher than that of those without dementia, and the patients with severe hearing loss suffering from dementia was 4.9 times of those with normal hearing.

In addition, our results suggested that the PTA has a significant correlation with orientation and abstract ability, indicating that hearing loss might affect the cognitive function by affecting orientation and abstract ability. Orientation is the ability of the individual to perceive signal and recognize the surrounding environment (time, place/space, and person) and one’s own state (name, age, occupation, etc.). Hearing loss causes the decline of hearing sensitivity and the speech perception in noise (Yamasoba et al., 2013), consequently affects the individual’s recognition of the surrounding environment and their own state, and ultimately leads to disorientation. It has shown that chronic hearing loss can lead to a decline of central auditory function (Peelle et al., 2011), activating the central compensation mechanism (Wild et al., 2012), and ultimately leading to the associated disorder of the auditory central-limbic system and the disuse atrophy of the frontal lobe (Wager et al., 2008; Lin et al., 2014). As we know, the frontal lobe is related to physical activities and mental activities such as judgment, predictability, emotion, and mood, which will reduce cognitive activity, increase the risk of depression, and lead to executive and emotional disorders. Liu et al. (2014) also found that the impairment of frontal lobe is more likely to be accompanied by the orientation and abstract disorders, which may be caused by the combined effects of nerve degeneration and vascular injury, which is consistent with the results of this study.

### The Correlation Between High-Frequency Hearing Impairment and Cognitive Function

Presbycusis, the hearing of the old man, has been recognized since the late 19th century (Kearns, 1977), and the high frequency is first affected. Studies have shown that degenerative changes in the inner ear of aged humans and other mammals occur among the sensory hair cells, the primary sensory neurons or spiral ganglion cells, and the cells of the stria vascularis and spiral ligament including the vasculature (Keithley, 2020). Neural degeneration is a very common pathology of the aged inner ear, both in humans and other animals, and occurs in both the apical and basal cochlear turns (Makary et al., 2011; Viana et al., 2015). In fact, the magnitude of neuronal loss exceeds that of inner hair cell loss in both humans and other mammals (Chen et al., 2006; Wu et al., 2019). So we deemed that the pure high-frequency hearing loss of the elderly was more likely to be caused by neural degeneration. In 2020, a study conducted by Liu et al. on APP/PS1 Alzheimer’s disease mice also found that the hearing loss appeared at high frequency as early as 2 months old, prior to the reported occurrence of spatial learning deficit at 6 to 7 months of age in this AD mouse model. The hearing loss was progressive and extended from high frequency to low frequency. We speculated that high-frequency hearing is more closely related to cognitive function (Liu et al., 2020).

The results of this study showed that pure high-frequency hearing loss has no obvious correlation with the general cognitive function, but was significantly related with the abstract and speech function; that is, early high-frequency hearing loss will affect the speech and abstract ability among the elderly. Some studies have already shown that the high-frequency sensitivity has a significant correlation with speech recognition ability, especially speech perception in noise (Monson et al., 2019),which is consistent with the results of this study. However, it is widely believed that the high-frequency hearing plays little or no role in speech perception, being beyond the information-bearing traditional “speech bandwidth.” Therefore, although the incidence of high-frequency hearing loss is significantly higher than that of speech frequency, there are still few studies on the correlation between pure high-frequency hearing loss and cognitive decline except for the speech perception. Jung and Haier (2007) hypothesized that the neural basis of human intelligence is attributable to activities in the frontal–parietal functional network. In their hypothesis, collected visual and auditory information is processed in the occipital and temporal cortices and then fed forward to the parietal cortex where structural symbolism, abstraction, and elaboration emerge (Apostolova et al., 2006). Therefore, once the auditory function is damaged, it will affect the subsequent abstraction and elaboration emerge process, which is consistent with our results.

## Limitations

Of course, this study also has some limitations. First, the 201 elderly volunteers were enrolled from the free clinic of 2019 Ear Day at Peking University People’s Hospital, which would cause selection bias, resulting in the higher proportion of hearing loss in this study. Second, the sample size of this study is relatively small, only 40 cases of pure high-frequency hearing loss, and 18 cases of normal hearing. Finally, in this study, we chose only a general cognitive assessment scale, the MoCA scale, for ease of operation and separate scales for each cognitive domain, and cognitive behavioral tests will be added in subsequent studies.

## Conclusion

Age, hypertension, years of self-reported hearing loss, and years of education are independent related factors for cognitive decline among the elderly. PTA was not an independent influencing factor of cognitive function, which may affect cognitive function by influencing orientation and abstract ability. Pure high-frequency hearing loss may affect the speech and abstract ability of elderly patients, but not significantly affect their global cognitive function. The early high-frequency hearing loss is not easy to be detected by the elderly, as it is relatively mild and does not significantly affect the cognitive ability. But as the result of this study, it may have a certain impact on the speech and abstract ability, which is necessary to be detected and prevented early.

## Data Availability Statement

The raw data supporting the conclusions of this article will be made available by the authors, without undue reservation.

## Author Contributions

TD, XM, JZ, MD, and LY contributed to the study conception and design. TD, XM, and JZ supervised this research and wrote the first draft of the manuscript. All authors contributed to the material preparation, data collection, and read and approved the final manuscript.

## Conflict of Interest

The authors declare that the research was conducted in the absence of any commercial or financial relationships that could be construed as a potential conflict of interest.

## Publisher’s Note

All claims expressed in this article are solely those of the authors and do not necessarily represent those of their affiliated organizations, or those of the publisher, the editors and the reviewers. Any product that may be evaluated in this article, or claim that may be made by its manufacturer, is not guaranteed or endorsed by the publisher.
